# Single Molecule Measurements of the Accessibility of Molecular Surfaces

**DOI:** 10.3389/fmolb.2021.745313

**Published:** 2021-12-01

**Authors:** Arpan Dey, Vicky Vishvakarma, Anirban Das, Mamata Kallianpur, Simli Dey, Roshni Joseph, Sudipta Maiti

**Affiliations:** Department of Chemical Sciences, Tata Institute of Fundamental Research, Mumbai, India

**Keywords:** single molecule conformation, single molecule photobleaching, single molecule quenching, SASA, hIAPP (human islet amyloid polypeptide)

## Abstract

An important measure of the conformation of protein molecules is the degree of surface exposure of its specific segments. However, this is hard to measure at the level of individual molecules. Here, we combine single molecule photobleaching (smPB, which resolves individual photobleaching steps of single molecules) and fluorescence quenching techniques to measure the accessibility of individual fluorescently labeled protein molecules to quencher molecules in solution. A quencher can reduce the time a fluorophore spends in the excited state, increasing its photostability under continuous irradiation. Consequently, the photo-bleaching step length would increase, providing a measure for the accessibility of the fluorophore to the solvent. We demonstrate the method by measuring the bleaching step-length increase in a lipid, and also in a lipid-anchored peptide (both labelled with rhodamine-B and attached to supported lipid bilayers). The fluorophores in both molecules are expected to be solvent-exposed. They show a near two-fold increase in the step length upon incubation with 5 mM tryptophan (a quencher of rhodamine-B), validating our approach. A population distribution plot of step lengths before and after addition of tryptophan show that the increase is not always homogenous. Indeed there are different species present with differential levels of exposure. We then apply this technique to determine the solvent exposure of membrane-attached N-terminus labelled amylin (h-IAPP, an amyloid associated with Type II diabetes) whose interaction with lipid bilayers is poorly understood. hIAPP shows a much smaller increase of the step length, signifying a lower level of solvent exposure of its N-terminus. Analysis of results from individual molecules and step length distribution reveal that there are at least two different conformers of amylin in the lipid bilayer. Our results show that our method (“Q-SLIP”, Quenching-induced Step Length increase in Photobleaching) provides a simple route to probe the conformational states of membrane proteins at a single molecule level.

## 1 Introduction

Solvent accessible surface area (SASA) is a frequently used parameter to probe the conformation of proteins (Momen-roknabadi et al., 2008; Kurt and Cavagnero 2006; Shrake and Rupley 1973). It is defined as the surface area of the molecule accessible to the solvent. For different states of folding of a given protein, this measures how compact and globular the conformation is (Mukherjee and Prasad Bahadur, 2018). The concept is simple, and SASA can be exactly calculated from an atomic level model of a given conformation. For this calculation, the solvent is typically represented by a sphere of a certain radius. What makes it powerful is that there are various ways that it can also be estimated experimentally (Shrake and Rupley 1973). A common estimate uses quenching of the fluorescence of a fluorophore attached to a given segment of a protein by a small solute molecule (Calhoun et al., 1986; Hashemi and Alizadeh 2009). Of course, it is not strictly “solvent” accessibility, but rather “solute” accessibility. However, if the quencher is a small solute, it provides a measure of the same quantity. Also, it only probes local accessibility near the fluorophore. Different parts of the protein need to be separately labelled to obtain a reasonable estimate for the SASA in this manner.

Fluorescence quenching is typically measured in the bulk, and provides an average measure of the population. The reduction of fluorescence is measured as a function of concentration of the quencher. The quenching can be due to a ground state complex formation (so called “static quenching”) or due to collisions in the excited state (so called “dynamic quenching”). Dynamic quenching can be separately measured from the fluorescence lifetime. Both processes report the accessibility of the fluorophore to the quencher. For example, this provides a useful measure of the change of conformation associated with the folding of a protein molecule (Midoux et al., 1984).

Dynamic quenching measurements, as measured by fluorescence lifetime, can in principle provide information about multiple conformational species present in the bulk solution. However, the ultimate resolution of such measurements would be achieved if one could measure the accessibility of each individual molecule (or molecular complex) in a sample, so that a complete population distribution of the measured property can be obtained. Such a technique would be very useful for understanding toxic amyloid oligomers, where a solution can contain oligomers of multiple stoichiometry, and each of those species can in principle have different conformations (Breydo and Uversky 2015). This would also be important in understanding conformational changes in intrinsically disordered proteins, where interaction with a ligand can partially condense the protein to one of its many accessible conformational states (Alam et al., 2019; Sahoo et al., 2020). Single molecule measurements in principle can provide such detailed information (Weiss 1999; Schuitz, Schindler, and Schmidt 1997). The power of single molecule techniques has been demonstrated in determining the dynamics of individual protein molecules (e.g. using smFRET (Mazal and Haran 2019; Huang Fang et al., 2009; Vogelsang, Sauer, and Tinnefeld 2007; Huang F. et al., 2009)), and single molecule quenching by intramolecular tryptophan (Hudgins et al., 2002; Neuweiler et al., 2003; Goluguri, Sen, and Udgaonkar 2019). While this provides kinetic information and is related to the protein conformation, it can only report the presence of two (or at best a few) discrete states. Single molecule quenching in the vicinity of a plasmonic metal particle has been used for measuring the distance of a fluorophore from the surface of the nanoparticle (Acuna et al., 2012; Chandra et al., 2018). Another technique, usually called “single molecule photobleaching” (“smPB”) has been shown to be an effective tool for determining the stoichiometry of small molecular aggregates (Gordon, Ha, and Selvin 2004; Ding et al., 2012; Dey et al., 2020a). However, none of these single molecule techniques directly reported solvent accessibility.

Here we develop a technique based on photobleaching and quenching to measure the accessibility of a fluorophore (which is covalently linked to a specific part of a protein) to a small solute, at the single molecule level. The strategy is to measure the time each molecule takes to photobleach. Since photobleaching occurs typically from the excited state (singlet or triplet) (Ha and Tinnefeld 2012), a quencher which can reduce the time that a molecule spends in the excited state would tend to increase the time it takes to photobleach (Widengren and Rigler 1996). Therefore, the accessibility of the dye-attached part of the protein can be quantitatively measured in terms of the relative increase of the photobleaching time. We named our method “Q-SLIP”, for Quenching-induced Step Length increase in Photobleaching. Our measurement provides the complete distribution of photobleaching step lengths, manifesting the inherent heterogeneity of the population. We validate the technique by measuring the accessibility of rhodamine-B fluorophore in two different molecular systems attached to lipid bilayers. The first is a dye directly attached to a lipid headgroup, the other are three dyes attached to a charged peptide which in turn is attached to a fatty acyl chain. We then demonstrate the power of the technique by measuring the solvent accessibility of a dye attached to amylin/human islet amyloid polypeptide (hIAPP, an amyloid peptide associated with Type II diabetes (Mirzabekov, Lin, and Kagan 1996)). How amylin attaches to the lipid bilayer is a matter of debate and our measurements shed light on this problem by showing that there are at least two different populations with different modes of attachment to the lipid bilayer.

## 2 Materials and Methods

### 2.1 Materials

#### 2.1.1 IAPP Preparation

Protocol for preparation of rhodamine-B labelled hIAPP (rh-hIAPP) is already described elsewhere (Rawat et al., 2018). hIAPP was synthesized using solid phase peptide synthesis (PS3; Protein Technologies, Tucson, AZ). Briefly, the peptide was synthesized on Rink Amide MBHA resin LL (100–200 mesh, loading capacity 0.35 mmole g-1, Novabiochem, Merck, Germany) leaving the C-terminal of the synthesized peptide amidated. 4-fold excess Fmoc protected amino acids were activated with equimolar HATU and NMM (0.4 M) in DMF. Subsequently, the Fmoc was deprotected using mixture of 2% DBU and 20% piperidine in DMF (v/v). Rhodamine-B labelling was performed on the N-terminus of the peptide (on resin). A mixture containing TFA, TIS, water, EDT, thioanisole, and phenol at a volume ratio of 32:1:2:1:2:2 was used to cleave the labelled peptide from the resin and deprotect the acid labile side chains (mixing time = 4 h). The TFA was removed from the peptide under nitrogen flow, and then precipitated and washed with tert-butyl methyl ether. The precipitate was dried under vacuum to obtain powdered crude peptides, which was purified using high performance liquid chromatography (HPLC, Shimadzu Prominence, Kyoto, Japan) and lyophilized. The mass spectra of the labelled peptide is provided in the SI (Supplementary Figure S1A). For further use, the lyophilized powder was stored at 4°C. For preparing stock solution, the powder was dissolved in an aqueous solution of pH 3.5 to a concentration of 1 mM. It was aliquoted and flash frozen for further use. For smPB experiment, an initial stock solution of IAPP was prepared in phosphate-buffered saline (PBS) (20 mM Na_2_HPO_4_ and 150 mM NaCl) at pH 7.5.

#### 2.1.2 Tris-Rhodamine Labelled Palmitoylated Peptide Preparation

Protocol for preparation of the tris-rhodamine-B labelled palmitoylated peptide is already described elsewhere (Dey et al., 2020b). A short peptide-based template with three lysines, H_2_N–KSQKTTKI–CONH_2_ was chosen. It was synthesized in an automated solid phase peptide synthesizer (PS3, Protein Technologies Inc., Tucson, AZ, United States) using the standard Fmoc (9-fluorenylmethoxycarbonyl) chemistry. Briefly, the peptide was synthesized on the same Rink Amide MBHA resin LL following the same protocol as mentioned above. Fmoc-Lys (Mtt)-OH (N-α-Fmoc-N-ε-4-methyltrityl-L-lysine) was used for the synthesis of the lysines. Hence all the lysine-side chains were orthogonally protected with the 4-methyltrityl (or ‘Mtt’) protecting group. After the synthesis of the full peptide, the last Fmoc on the N-terminal of the peptide was retained while the Mtt-groups were deprotected using 2% TFA in dichloromethane (DCM) with 5% triisopropylsilane (TIS). Three rhodamine-B dyes were covalently attached to the three lysines. For selectively labelling the three lysines while keeping the N-terminus of the peptide unlabelled, the orthogonal deprotection chemistry was used. The final sequence of the tris–rhodamine-B labelled template was Fmoc-HN-K(Rh)SQK(Rh)TTK(Rh)I-CONH_2_. For lipidation, the N-terminal f-moc was removed using 20% piperidine in dimethyl formamide (DMF). 10 M equivalents of activated palmitic acid (activation using hexafluorophosphate azabenzotriazole tetramethyl uronium, HATU) and 20 M equivalents of diisopropylethylamine (DIPEA) were used for the ligating fatty acid chain to the N-terminal amine of the peptide. The sequence of the final fatty acid-tailed tris labelled peptide was palmitoyl-HNK(Rh)SQK(Rh)TTK(Rh)I-CONH_2_. The mass spectra of the tris-rhodamine-B labelled palmitoylated peptide is provided in the SI (fig SI-1(B)) All Fmoc amino acids and reagents were purchased from Merck (Germany). Palmitic acid was purchased from Sigma-Aldrich (St Louis, Missouri, United States). The fatty acid-tailed labelled peptide was further purified using HPLC. Finally, matrix-assisted laser desorption/ionization–time of flight (MALDI–TOF) mass spectrometry was used for characterization.

#### 2.1.3 Lipid Vesicle and Supported Lipid Bilayer Preparation

Lipids vesicles were prepared using the sonication method (Dey et al., 2020a). The lipids 1-palmitoyl-2-oleoyl-sn-glycero-3-phosphocholine (16:0–18:1, PC) (POPC), 1-palmitoyl-2-oleoyl-sn-glycero-3-[phospho-rac-(1-glycerol)] (16:0–18:1 PG) (POPG) and 1,2-dioleoyl-sn-glycero-3-phosphoethanolamine-N-(lissamine rhodamine B sulfonyl) (ammonium salt) (18:1 Liss Rhod PE/rh B-PE) were purchased from Avanti Polar Lipids (Alabaster, AL) and Cholesterol (Chol) from Sigma-Aldrich (St Louis, MO). Prior to vesicle formation, a lipid film was prepared. For preparing PPC (1:1:1) bilayer, POPC, POPG and Cholesterol were taken in a pre-cleaned glass vial in molar ratio 1:1:1 and dissolved in HPLC graded chloroform (S.D. Fine Chemicals Ltd., India). After complete dissolution of the lipids, chloroform was evaporated by purging Argon with slow rotation until the lipids formed a uniform thin film around the wall of the vial. The lipid film so prepared was kept in a vacuum chamber overnight for complete removal of traces of chloroform. For preparing vesicles, the lipid film was rehydrated using deionized water to a final concentration of 5 mg/ml. The turbid suspension so produced was vortexed and sonicated rigorously at 60°C till a clear solution of lipid vesicle formed. For preparing bilayer, the lipid vesicle solution so produced was put in a chamber made of precleaned coverslip (both piranha and plasma treated) and plastic PCR tubes. 10 mM CaCl_2_ solution was added to the vesicle solution. The entire assembly was kept in a water bath at 60°C for an hour and slowly allowed to cool. This facilitated formation of the lipid bilayer. After the bilayer formed, excess unfused vesicles were removed by gentle deionized water wash. For preparing bilayer with rh B-PE for confocal bleaching experiments, it was added in a mole ratio of 1:10 (labelled lipid vs unlabelled PPC (1:1:1) lipid was added in the ratio1:10).

#### 2.1.4 smPB Sample Preparation

For preparing samples for smPB, the fluorescent markers should be stationary. This allows correct monitoring of the bleaching steps. Hence, to immobilize the fluorescent spots, either samples were spin coated or attached to/distributed on a lipid bilayer. For spin coating, samples were diluted in pH 7.5. In 0.25% poly vinyl alcohol (PVA) solution, the samples were added to prepare a final concentration of 0.5–1 nM. On precleaned coverslips (both piranha and plasma treated), PVA sample solution was spin coated (3000 RPM) for 30 s. This ensured uniform coating and well dispersed fluorescent spot density for imaging. For monitoring effect with tryptophan, 5 mM tryptophan was also added into the prepared PVA solution before spin coating. The tris–rh-labelled-lipidated-peptide/rh B-PE was incorporated in the lipid during the preparation of the lipid film. For monitoring smPB of tris–Rh-labelled-lipidated-peptide, it was added to the lipids in a molar ratio of 1:10^7^ of labelled: unlabelled POPC, POPG and Cholesterol (1:1:1). Similarly, for rh B-PE, about 500 pM of labelled lipid was added along with unlabelled lipid. Then the bilayer was prepared using the protocol described above and imaged using a total internal reflection fluorescence (TIRF) microscope (Axelrod 1981; 2016). For imaging the tryptophan induced effect, 5 mM tryptophan was added on top of the bilayer and imaged. For monitoring hIAPP oligomers, solution of hIAPP oligomers (pH 7.5) was added on the bilayer to a final concentration of 0.5–0.9 nM. After incubation (30 min), the excess unattached or loosely attached oligomers were washed off. The incubation time depended upon the type of experiment being monitored. After imaging several fields, 5 mM tryptophan solution was added to monitor tryptophan-induced changes if any.

### 2.2 Methods

#### 2.2.1 Quenching-Induced Increase in Photobleaching Step Length

**FIGURE 1:**
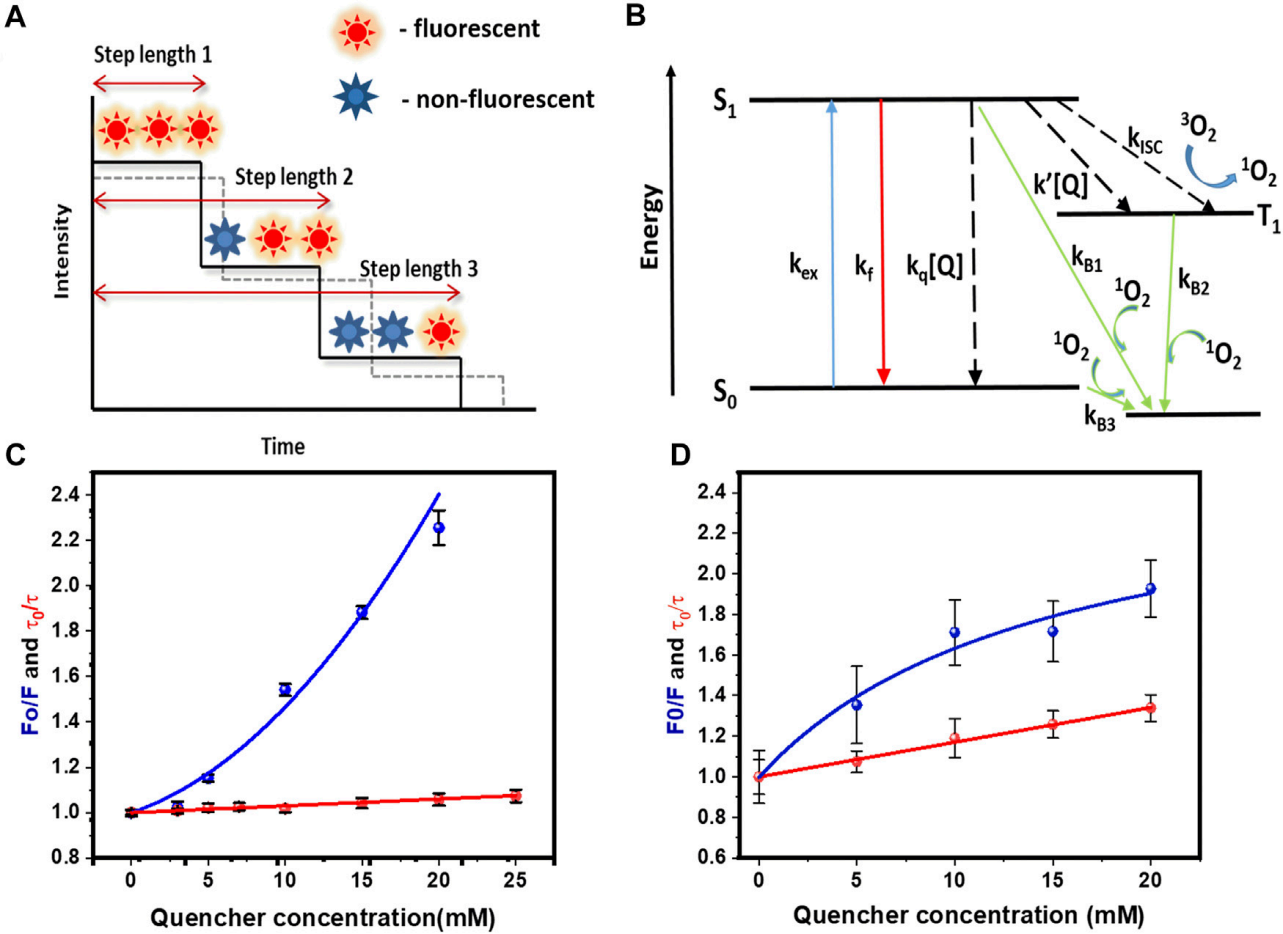
Quenching of rhodamine-B by tryptophan, and its effects on fluorescence, lifetime and single molecule photobleaching, **(A)** Cartoon representation of bleaching step lengths obtained from a single molecule photobleaching trajectory. Dashed line shows increase in step length in presence of a quencher. **(B)** Schematic showing how quenchers can modulate various processes affecting the rate of photobleaching. k_ex_, k_f_, k_q_ [Q], k_isc_, k’ [Q] are excitation, fluorescence, dynamic quenching, intersystem crossing, and quencher induced inter system crossing rates. Quenchers can increase or decrease the photobleaching rates **(C)** Stern-Volmer plot of steady state (blue) and dynamic (red) quenching measurements of rhodamine-B in solution by tryptophan. F_0_ (F) = fluorescence without (with) 5 mM tryptophan. τ_0_ (*τ*) = lifetime without (with) 5 mM tryptophan. Values are mean ± SEM. **(D)** Stern-Volmer plot of steady state (blue) and dynamic (red) quenching measurements of rh-B-PE in PPC 1:1:1 vesicles by tryptophan. Values are mean ± SEM.

From the single molecule photobleaching trajectory of a fluorescent spot (Arant and Ulbrich 2014), two types of information can be obtained. First, the number of fluorophores present in a spot (characterized by the number of bleaching steps), and second, time taken by each fluorophore to bleach. This is called “step-length”, and can be measured by monitoring the time taken by the fluorophore to photobleach from the time “zero” (start of irradiation). In this work, we have used fluorophores which are attached to molecules that gets embedded into a lipid bilayer. This allowed us to individually track the brightness of each particle in time. A single-step decrease in the brightness signified the bleaching of a single fluorophore. Figure 1A shows a schematic representation of this process. Photobleaching is positively correlated to the time a fluorophore spends away from the ground state. Therefore, any process that can return the molecule from the excited state to the ground state in principle should reduce the photobleaching probability (and hence increase the time step-length). Therefore, presence of fluorescence quenchers is expected to increase the step length, provided the quencher has access to the fluorophore (Figure 1A, shown by a dotted line).

This is the principle we have used in this work. We have measured the fractional change in the photobleaching step length as a function of quencher concentration. For a given concentration of the quencher, the larger the relative increase in step length (relative to zero quencher concentration), the more exposed is the fluorophore. We have used rhodamine B as the labelling fluorophore, and tryptophan as the quencher. Tryptophan is a general quencher used in literature for probing conformational fluctuations in proteins because of its quenching abilities (Ahsan et al., 2010; Goluguri, Sen, and Udgaonkar 2019). Tryptophan shows a similar type of quenching (both static and dynamic) with fluorescein also (fig A) details of which is provided in the Supplementary Figure S2A–C). The actual process of tryptophan-induced fluorescence quenching can be complex, and it has been discussed by several authors (Neuweiler et al., 2003; Sun, Lu, and Yu 2012; Widengren and Rigler 1996). We will only describe it briefly here. A minimal description requires three states, the ground electronic state (S_0_), first excited electronic state (singlet S_1_), and the excited triplet state (T_1_) (Figure 1B). Upon excitation from S_0_ to S_1_, the S_1_ state can get depopulated by returning to the S_0_ state radiatively (fluorescence), or non-radiatively. Also, it can undergo an inter system crossing (ISC) and populate the T_1_ state. Interaction between this long-lived triplet state and triplet oxygen present in water can generate reactive singlet oxygen (^1^O_2_). ^1^O_2_ can oxidize the nearby fluorophore and render it non-fluorescent. Since the ^1^O_2_ concentration in the vicinity of a fluorophore is strongly correlated with photo-excitation (via the T_1_ state), the rate of photobleaching depends on the probability of the fluorophore being in the excited state. Collision with a quencher can increase the rate of non-radiative S_1_ to S_0_ transition (K_q_[Q] as shown in Figure 1B). Additionally, processes like ground state complex formation with the quencher can prevent the S_0_ to S_1_ transition altogether. Either way, the probability of the fluorophore being in the excited state is reduced, and the probability of photobleaching goes down, increasing the bleaching step-length. We note that there is a possibility that tryptophan can increase the ISC process, thereby increasing the probability of photobleaching. However, this system does not show an altered triplet relaxation rate (as measured by fluorescence correlation spectroscopy, data not shown), and so we do not consider that possibility. We also note that the ground state complex formation itself can be a dynamic process, so that the fluorophore is occasionally free of the quencher, making it bright enough to be visualized at the slower time scale of fluorescence imaging. The first process will be reflected in a reduced lifetime of the fluorophore, and the second process by a reduced brightness. A plot of F_0_/F vs quencher concentration (called the “Stern-Volmer” plot (Patil et al., 2011), where F_0_ is the steady-state fluorescence at zero concentration of the quencher, and F is the fluorescence at a given quencher concentration) can quantify the quenching process. A separate measurement of the lifetime can reveal how much of the quenching is due to fast collisional processes, and how much is due to ground state complex formation.

#### 2.2.2 Steady State and Time Resolved Quenching Measurements

Steady state fluorescence quenching measurements of rhodamine-B dye and Rh-PE in PPC (1:1:1) vesicles by tryptophan were carried out in a standard fluorimeter (Fluoromax-3, Jobin Yvon Horiba). For lifetime quenching measurements, time-resolved fluorescence decay were performed for rhodamine-B dye and Rh-PE:PPC (1:1:1) vesicles with different concentration up to 50 mM of tryptophan. All samples were excited with a Rhodamine 6G dye laser that generated pulses of < 50 ps width. Samples were excited at 570 nm in a quartz cuvette (1 × 1 cm path length). The emission was collected using a Multi-Channel Plate Photo Multiplier Tube (MCP PMT). A neutral density filter was used to control the intensity off the excitation pulse so that the emission rate is less than 1/100th time of the excitation rate. Also, a 590 nm long pass glass filter was used to cut off the excitation photons. Signal was collected at 600 nm using a monochromator. The signal was acquired at a magic angle with respect to the excitation polarization. Each measurement was continued till a maximum of 10,000 photons was achieved. Each time, the Instrument Response Function (IRF) was measured using a dilute solution of non-dairy coffee whitener. While measuring the IRF, the long pass filter was removed and the signal was collected at 565 nm. The Full Width Half Maxima (FWHM) measured was always less than or equal to 100 ps.

#### 2.2.3 Ensemble Bleaching Measurements on a Bilayer

We measured the bleaching kinetics of rh-B PE lipid in a supported lipid bilayer (PPC 1:1:1) in presence and absence of tryptophan. Continuous spot photobleaching in an area containing fluorophore reduces the fluorescence intensity gradually, and the kinetics of this decrease reports on the rate of photobleaching (other parameters such as diffusion remaining constant (Balaji, Sengupta, and Maiti 2001)). The rh-B PE lipid containing bilayer was subjected to a confocal laser scanning microscope (LSM710, Zeiss, Germany and LSM880, Zeiss, Germany) to monitor its decay characteristic. In the confocal image (using a 40× oil immersion objective (1.2 NA)), a field of view at a particular z plane was chosen for bleaching. The field of view was zoomed to an area of 5.3 µm × 5.3 µm. The back aperture intensity was kept at 35 µW. Each frame recorded were an average of 16 scans. We note that photobleaching in two dimensions does not approach a steady state (Balaji, Sengupta, and Maiti 2001). We continued the experiment till the bleaching was nearly complete (about 20 min). For monitoring the effect of tryptophan, similar measurements were performed with 5 mM tryptophan added to the medium containing the bilayer.

#### 2.2.4 smPB Measurements and Data Analysis

**FIGURE 2:**
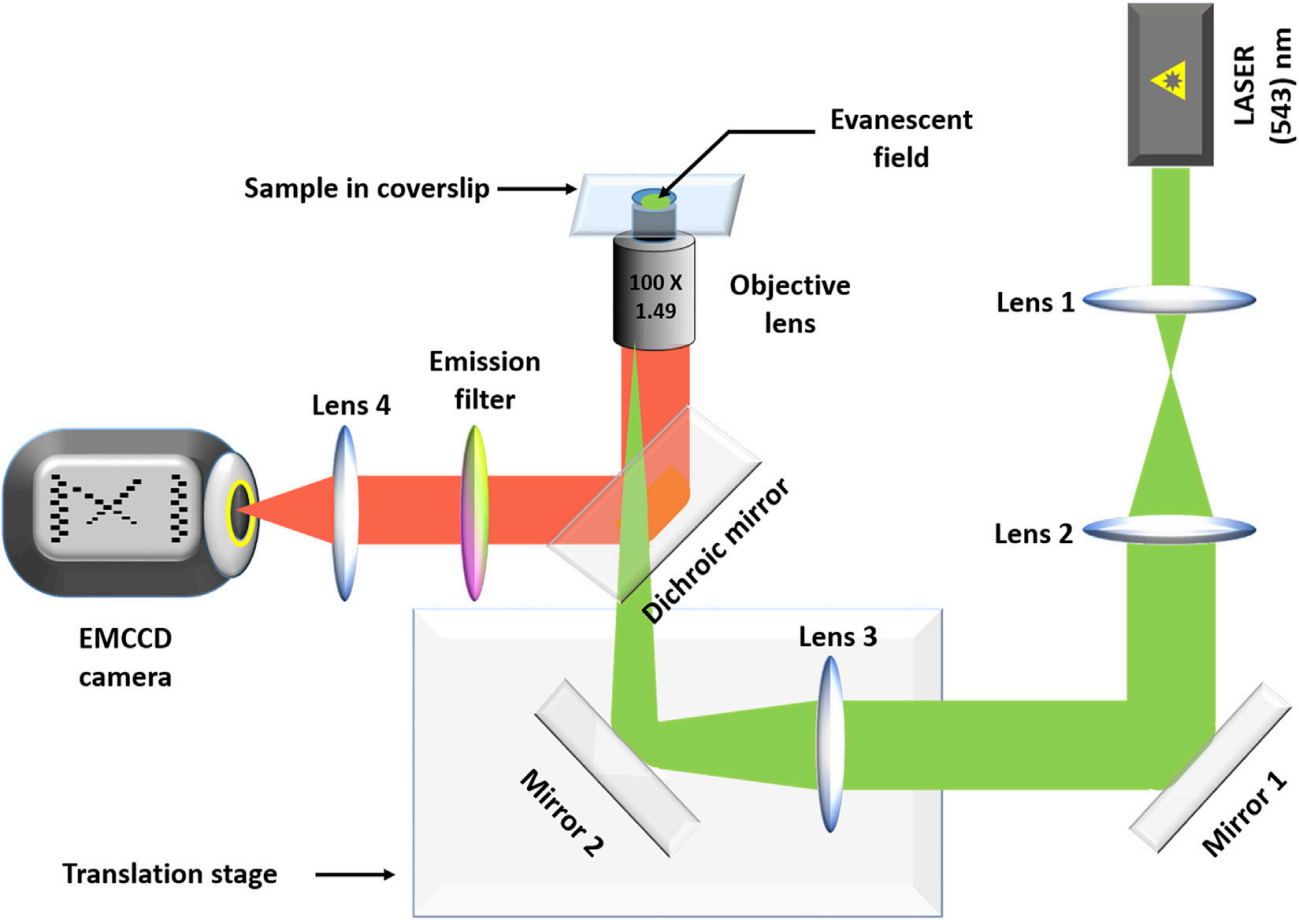
The home-built TIRF set up. Lenses 1 and 2 form a telescope that increases the diameter of the excitation laser beam (green). Lens 3 focuses the beam at the back focal plane of the objective lens. The translation stage is used to adjust the penetration depth, as needed. The fluorescence (red) is separated from the excitation beam using a dichroic mirror. The fluorescence is passed through an emission filter before it is focused on to an EMCCD camera using the tube lens (lens 4).

All the smPB images were acquired using a home-built objective lens based TIRF microscope using a high numerical aperture objective lens (NA 1.49, 100; Nikon, Tokyo, Japan). A Prism-based TIRF system would have a better uniformity of illumination, but that is not a primary concern for a step-photobleaching experiment. On the other hand, changing bilayer samples would have been somewhat more complicated in a prism-based system. The instrumentation has been discussed elsewhere (Dey and Maiti 2018). Briefly, a 543-nm He-Ne (25-LGR-393–230; Melles Griot, Rochester, NY) laser was used for excitation. A dichroic (565 nm) was used to separate the excitation from the fluorescence. The fluorescence was collected using a band-pass filter (605/55 nm, BA577-633, Nikon) and focused into an electron multiplying CCD camera (ANDOR iXON, DV887ECS-UVB). For identifying stoichiometry of the spots or the effect of tryptophan on photostability movies were captured for time durations long enough to bleach the fluorescent spots. The frame rate was kept at 90 ms. A diagrammatic representation of the entire set-up is given in Figure 2.

We used the TrackMate plugin in Fiji (Tinevez et al., 2017) to track the fluorescent spots. While we identified the spots using Trackmate, both stoichiometry and step length of the spots were calculated manually. Although there are various automated step counting algorithm, in our experience their performance is very sensitive to the quality of individual datasets. Thus we preferred to perform a manual detection and estimation of the data. A minimal diameter of four pixels were chosen for the spots. A threshold was always selected for each image for subtracting the background. Also a control subtraction without the molecule of interest was always performed. Since in most of the experiments, the fluorescent molecules were either encased in PVA matrix or attached to membrane, they were immobile and showed minimal movement. Only spots that were visible for more than 10 frames were considered for analysis. The spots showing long trajectory movements or overlapping tracks were neglected. Also, spots having overlapping point spread functions were not considered.

## 3 Results

### 3.1 Tryptophan Induced Quenching in Solution and in Lipid Bilayers

We first characterized tryptophan induced quenching of rhodamine-B using ensemble experiments in solution and in lipid bilayer vesicles. We used steady state fluorescence and Time Correlated Single Photon Counting (TCSPC) to monitor static and dynamic quenching by tryptophan. The fluorescence lifetimes “τ” were determined at different tryptophan concentrations, and the data was fitted using the Stern-Volmer equation (Eq. 1),

$$\frac{\tau_{0}}{\tau }=1+k_{q}\tau_{0}\left[Q\right]$$ (1)

where τ_0_, τ, K_q_, and [Q] represents the lifetime in absence of quencher, lifetime in presence of quencher, bimolecular quenching constant, and quencher concentration respectively. From the analysis, we found that tryptophan has a bimolecular quenching rate k_q_ of 1.6 × 10^9^ M^−1^s^−1^ for rhodamine-B (Figure 1C). We also measured the steady state fluorescence of rhodamine-B at different concentrations. The Stern-Volmer plot showed a non-linear response and was fitted using an equation that considers a “sphere of action quenching” (Doose, Neuweiler, and Sauer 2005),

$$\frac{F_{0}}{F}=\left(1+k_{q}\tau_{0}\left[Q\right]\right)e^{\left[Q\right]K_{s}}$$ (2)

Here, we found tryptophan has an exponential contribution to the static quenching with a static quenching constant K_s_ = 38.3 ± 2.1 M^−1^. This indicated both static and dynamic processes are involved in the quenching of rhodamine-B by tryptophan [Figure 1C].

We also performed quenching experiments in lipid bilayers, using both steady state and fluorescence lifetime measurements. We prepared vesicles from lipid film (PPC 1:1:1) containing 0.0001 mol% of rhodamine-B labelled PE lipid (rh-B PE). The stern-Volmer plot obtained for the steady state quenching with tryptophan was fitted using the following equation,

$$\frac{F_{0}}{F}=\;\frac{F_{0}^{a}+F_{0}^{b}}{\frac{F_{0}^{a}}{1+K_{a}\left[Q\right]}+F_{0}^{b}}$$ (3)

Here, F_0_^a^ and F_0_^b^ represents the accessible and inaccessible fluorophore populations respectively. Thus in presence of quencher, the fluorescence of “b” remains unaffected, but “a” gets quenched. K_a_ is the Stern-Volmer quenching constant for the accessible fraction “a” (Lackowicz, 1983). Steady state measurements showed a sub-linear response (Figure 1D). This is likely due to the presence of a heterogeneous population with different accessibility of rhodamine-B headgroups to tryptophan (Lakowicz and Delman, 1980; Morris, Bradley, and Blumenthal 1985). The vesicle system shows a bimolecular quenching constant of 6.18 × 10^9^ M^−1^s^−1^ (Figure 1D). This unusually high value can be due to electrostatic interaction between the zwitterionic tryptophan and the negatively charged PPC bilayer which can increase the local quencher concentration. It is also possible that a part of the tryptophan population is incorporated in the membrane, and a two dimensional reaction-diffusion process contributes to this quenching (Vanderkooi and Callis 1974). In any case, these results from ensemble studies showed that tryptophan-induced quenching of a rhodamine-B labelled molecule incorporated in a lipid bilayer is modulated by its accessibility.

### 3.2 Ensemble Measurements of Quenching-Induced Photo-Stability in a Supported Lipid Bilayer (SLB)

**FIGURE 3:**
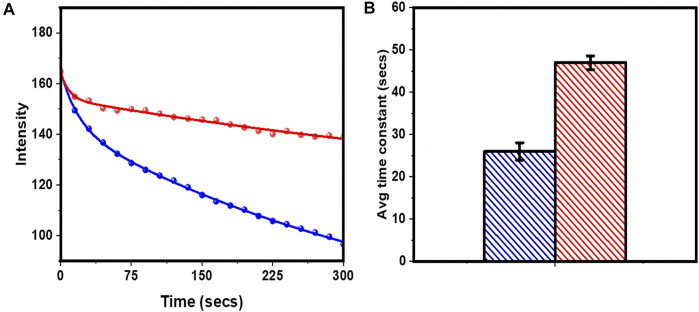
Localized photobleaching of rh-B PE in PPC 111 bilayer measured in a confocal microcsope. **(A)** Decay of fluorescence due to photobleaching without (blue cricles) and with (red circles) 5 mM tryptophan. Bi-exponential fits (Eq. 4) of intensity decay are shown as continuous lines of corresponding colours. **(B)** the average bleaching times constants (Eq. 5). Same colour code as in **(A)**. Values are mean ± SEM.

We then probed whether tryptophan can increase the photostability of rhodamine-B in bulk. We prepared a lipid bilayer (POPC:POPG:Cholesterol 1:1:1) with 18:1 Lissamine Rhodamine-B PE (rh-B PE) in the molar ratio of 10:1. We selected different regions on the bilayer and monitored its bleaching. The bleaching of the bilayer was monitored frame-wise (frame rate 15 s/frame) till it was completely bleached. 5 mM tryptophan was added to the same bilayer and its bleaching characteristic was monitored to check the effect of tryptophan on the rate of bleaching. The intensity decay is averaged over four different regions of two such prepared bilayers. A bleaching process coupled to diffusion in two dimensions does not approach a steady state (Balaji, Sengupta, and Maiti 2001). Here the initial parts (the first 20 frames) of the decay profiles (Figure 3A) were phenomenologically fitted using a bi-exponential equation

$$y=A_{1}e^{\left(\frac{-t}{T1}\right)}+A_{2}e^{\left(\frac{-t}{T2}\right)}+y_{0}$$ (4)

The average bleaching time was calculated as

$$\frac{\left(A_{1}T_{1}^{2}+A_{2}T_{2}^{2}\right)}{A_{1}T_{1}+A_{2}T_{2}}$$ (5)

From the fitted parameters, the average bleaching times were obtained as 26.0 ± 2.0 s and 47.0 ± 1.6 s (before and after adding tryptophan) respectively (Figure 3B). Thus 5 mM tryptophan decreased the rate of photobleaching of rhodamine-B by ~ 81% in a supported lipid bilayer. This showed that tryptophan can increase the photostability of rhodamine-B in the bulk.

### 3.3 Solute Accessibility of Individual Molecules in a SLB

**FIGURE 4:**
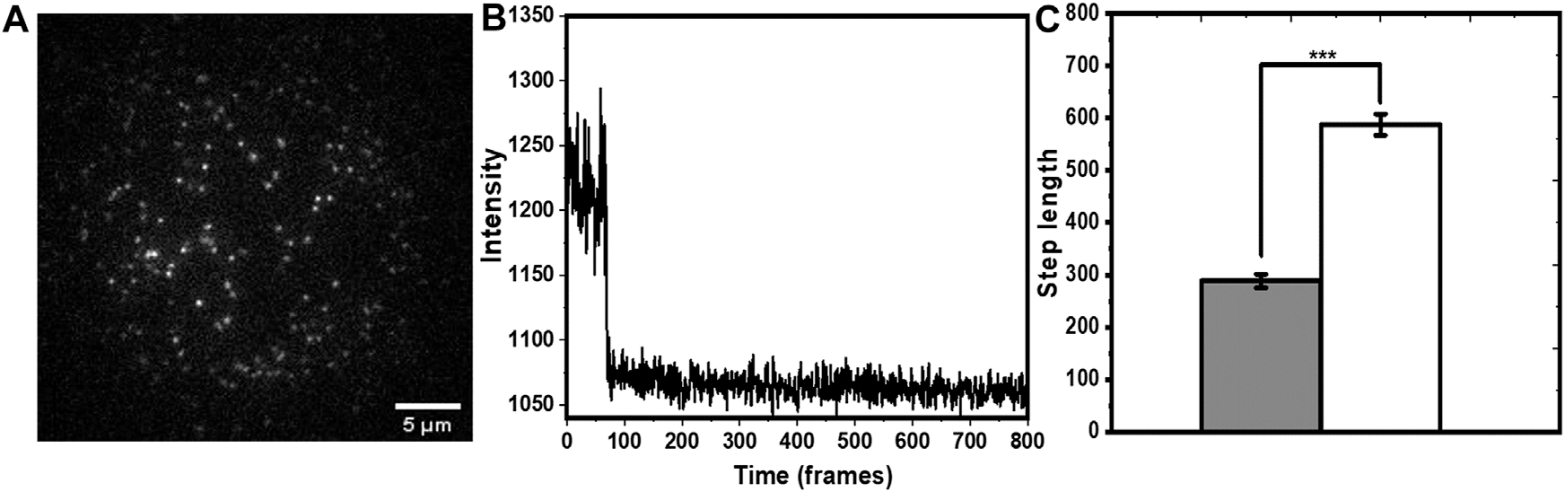
Single molecule photobleaching of rh-B PE in PPC 111 bilayer measured in TIRF. **(A)** A typical frame of a time-lapse image showing individual molecules as fluorescent spots. **(B)** Representative single step bleaching trajectory of a rh-B calculated from the time-lapse images. **(C)** increase in the bleaching step length (in units of frame numbers, each frame is 90 ms) of rh-B PE after tryptophan addition (grey and white represent step lengths before and after addition of tryptophan, respectively. *p* < 0.001, error bars are SEM).

We performed smPB of rh-B PE lipid in PPC (1:1:1) bilayer to check whether tryptophan induced decrease in photobleaching rate can be used to determine solvent exposure of single molecules. Rh-B PE was mixed with PPC (1:1:1) film to a final concentration of 50 pM. A typical field of view (Figure 4A) shows well separated spots of Rh-PE lipid attached to the bilayer. The spot intensities showed minimal movement with time, and their time traces showed a single step bleaching (Figure 4B). Although, a lot of mobility was observed in the rh-PE system (Pinkwart et al., 2019), for ease of analysis, we picked those fluorescent spots which were relatively less mobile (atleast for 10 frames). In principle, the moving spots can also be tracked for photobleaching and trajectories could be isolated, we avoided those complicated analyses. But this apparently does not have a consequence on the applicability of the technique. This was consistent with our expectation, since each rh-PE molecule had only one fluorophore. A few spots showed two-step events, most likely were from overlapping point spread functions (psf’s) of two independent molecules, and were neglected. We calculated the average bleaching step length of rh-B PE from about 200 such spots. It was found to be 288.5 ± 12.9 frames (25.9 ± 1.2 s). Upon addition of 5 mM tryptophan, the step length increased to 587.5 ± 20.3 frames (52.9 ± 1.8 s) (i.e., by a factor of 2.03 ± 0.15) (Figure 4C). The step lengths were represented in frame numbers. For converting it into time, the frame numbers were multiplied with 90 ms (time/frame). This value was similar to that obtained from the ensemble measurements although without any information of intrinsic heterogeneity. This demonstrated that tryptophan quenching combined with smPB can report the relative solvent accessibility of individual molecules.

**FIGURE 5:**
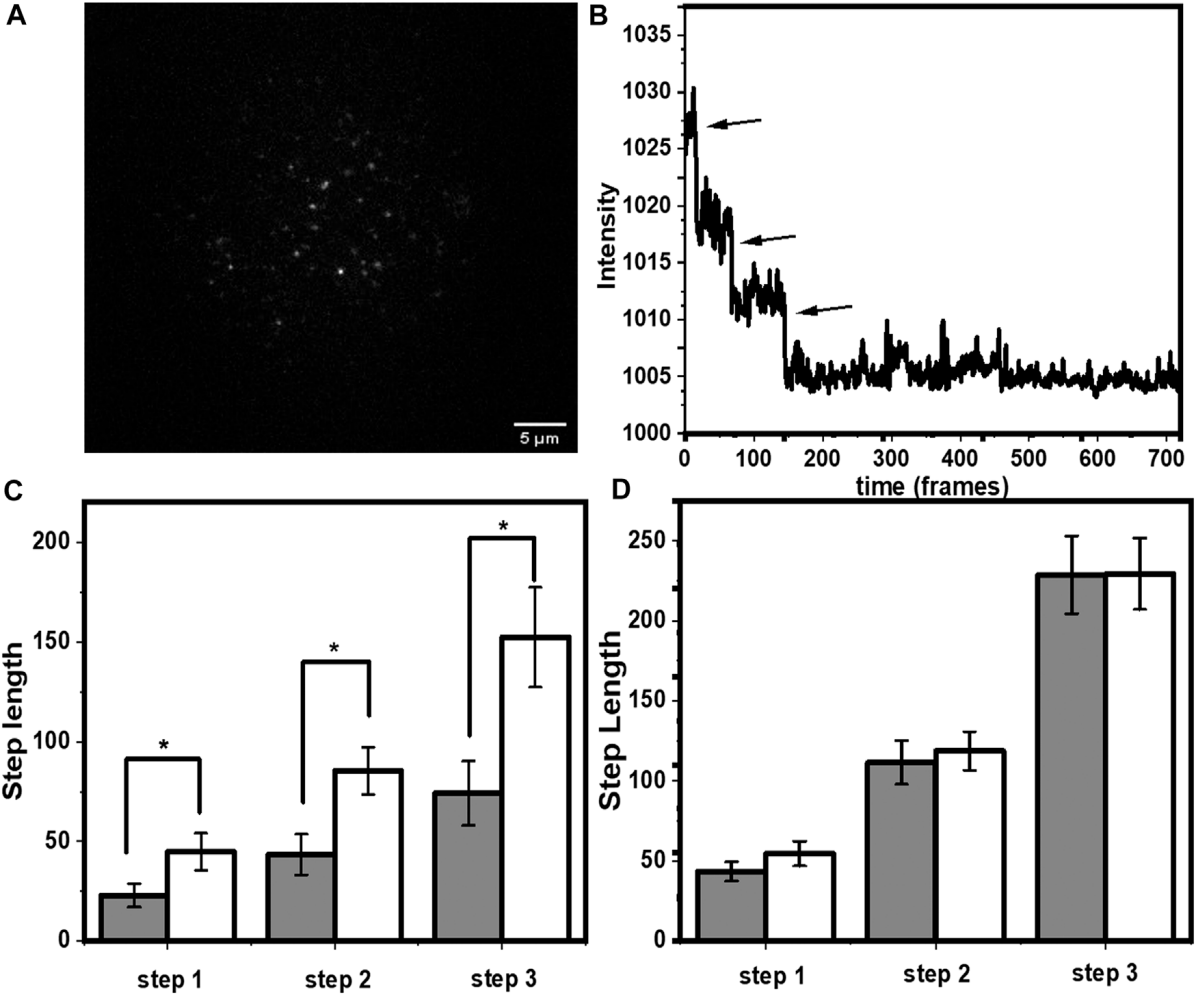
Single molecule photobleaching of lipid-tailed tris-rhodamine B labelled peptide. **(A)** A typical frame of a time-lapse image showing individual molecules as fluorescent spots. **(B)** Representative three step bleaching trajectory. **(C)** increase in step length (in units of frame numbers, each frame = 90 ms) for all the three rhodamine-B fluorophores after tryptophan addition. Grey and white represent step lengths before and after addition of tryptophan, respectively. *p* ≤ 0.05, error bars are SEM) and **(D)** Same molecule in PVA gel. Grey and white represent step lengths before and after addition of tryptophan, respectively. Error bars are SEM.

The power of single molecule photobleaching measurements lies in its ability to separate the properties of different oligomeric complexes. We probed a covalently-linked tris-labelled peptide-lipid complex, specifically constructed for such measurements (Dey et al., 2020b). This is a short, lipidated peptide H_2_N-KSQKTTKI-CONH_2._ All the three lysines of the peptide were labelled with rhodamine-B and the N-terminus was lipidated with palmitic acid, resulting in the molecule palmitoyl-HN-K (rh)SQK (rh)TTK (rh)I-CONH_2_. The three rhodamine-B dyes on each of the peptides allowed us to recognize the dye-stoichiometry of the molecule, from its characteristic three step bleaching. In a typical field of view (Figure 5A), not all the spots appeared trimeric, because of pre-bleaching (Dey et al., 2020a). We selected ~ 210 spots which appeared as trimers (Figure 5B) and compared the step lengths of all the individual spots for both before and after adding 5 mM tryptophan on the bilayer (Figure 5C). Tryptophan increased the photostability of all the three rhodamine-B molecules. The increase in step lengths were factors of 1.96 ± 0.89, 1.97 ± 0.74, and 2.05 ± 0.78 for the first, second and third steps respectively (mean ± SEM). This value was similar to the rh-PE measurements. This was also consistent with our expectation that the rhodamine-B labelled peptide would be in proximity of the headgroup region of the lipid. However, the large standard error indicated possible heterogeneity in the system. Also, we monitored the brightness of all the three rhodamine-B from the spots that showed a three step bleaching both before and after adding tryptophan. Since the photo-stabilization is because of an interaction with tryptophan (non-radiative decay or ground state complex formation), it is expected to affect the brightness of the fluorescent spots. Indeed, as described in the (Supplementary Figure S3A), the decrease in brightness is in accordance to the increase in photo stability.

We expected both the static and the dynamic quenching processes to contribute to the increase in the step length as the contribution from the dynamic collision process, reflected in the decrease in the fluorescence lifetime, was relatively modest. The static quenching process can only contribute if it is not completely static, i.e., the ground state complex formation is reversible in the time scale of imaging (90 ms). This can be tested by arresting the diffusion of tryptophan and rhodamine-B in solution. If tryptophan is not able to diffuse, the solution would either have a completely quenched rhodamine-B complex, or a free rhodamine-B. While the first species would not be observable, we would expect to see minimal effect of tryptophan on the bleaching step length. Thus, as a negative control, we probed an un-lipidated peptide with the same sequence, and with three rhodamine-B labels: H(Rh)N-QK(Rh)TTK(Rh)I-CONH_2_. The peptide was dissolved in 0.25% PVA (which rapidly polymerizes to inhibit diffusion), and the entire solution was spin coated on a cover slip. The cover slip was blow dried after coating. This formed a rigid matrix and reduced diffusion to a minimum. As seen in Figure 5D, we observed no significant increase in the step length for any of the three rhodamine-B molecules. Thus, we concluded that the step-length increase is dependent on reversible encounters with a diffusing quencher.

Although the above results show that quencher induced photo-stabilization can be efficiently used even at the single molecule level to study conformational accessibility of molecules, the data further allows a detailed understanding of the heterogeneity of the system. This is obtained by analyzing the difference in step length distributions of the individual fluorophores upon tryptophan addition. This is discussed in detail in section 3.4.

### 3.4 Accessibility of a Membrane Attached hIAPP Peptide

**FIGURE 6:**
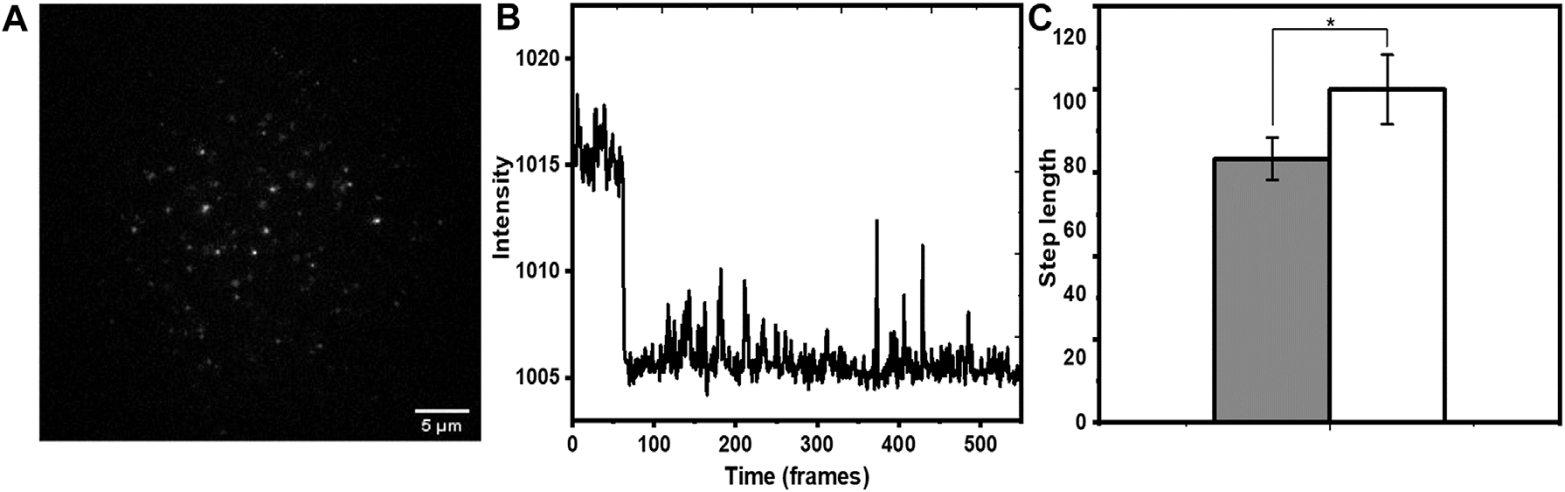
Single molecule photobleaching of N-terminus-rh-labeled hIAPP monomers on membrane. **(A)** A typical frame of a time-lapse image showing individual molecules as fluorescent spots. **(B)** Representative bleaching trajectory. **(C)** increase in step length (in units of frame numbers, each frame = 90 ms) after adding tryptophan (5 mM). Grey and white represent step lengths before and after addition of tryptophan, respectively. *p* ≤ 0.05, error bars are SEM.

We then employed this method to investigate the solvent exposure of membrane-associated hIAPP. hIAPP is an amyloidogenic 37 amino acid peptide which can interact with cell membranes (Rawat et al., 2018), and this interaction is suspected to play a major role in its toxicity. However, very little is known about the mode of its interaction with the membrane. An idea of the solvent exposure of different parts of hIAPP can help solve this puzzle. Thus, we compared the exposure of the N-terminus of the peptide (labelled with rhodamine) relative to that of rh-PE. In principle, dye labelling can change membrane attachment properties. Indeed, organic dyes commonly show some membrane affinity (Hughes et al., 2014). However, we have earlier shown that N-terminal labelling of hIAPP peptide with rhodamine does not affect its attachment properties (Rawat et al., 2018). We incubated a sub nM solution of rh-hIAPP with the PPC 1:1:1 bilayer. A typical field of view (Figure 6A) showed well separated fluorescent spots on the bilayer. Those hIAPP spots that bleached in a single step (Figure 6B) were chosen for analysis. In the case of hIAPP, we found more immobilized species on the bilayer. Early reports (Chang et al., 2018) also reported similar immobilized amyloid oligomers. They provided a plausible explanation that oligomers span the entire bilayer and comes into direct contact with the glass substrate. We possibly observe very similar phenomena. We measured the increase in step lengths for the monomers (695 spots) induced by 5 mM tryptophan (Figure 6C), and found a relative increase by a factor of 1.26 ± 0.23. This was much less compared to the average values for both rh-B PE in the bilayer, as well as the bilayer-anchored lipidated tris rhodamine-B peptide. This implied that the N-terminus of hIAPP is much less exposed to the solution than the rhodamine-B molecule attached to the headgroup of PE (we note that the error bars were large, possibly again indicating some heterogeneity in the population). This was also observed for the lipidated peptide, though the error bars were much smaller for rh-PE. To analyze this heterogeneity in terms of the underlying sub-populations, we plotted a distribution of the step lengths for all the system both before and after tryptophan quenching, and fitted them to a single, or a sum of two exponentials (Eq. 6).

$$y=A_{1}e^{\left(\frac{-t}{T1}\right)}+A_{2}e^{\left(\frac{-t}{T2}\right)}$$ (6)

Here, “A_1_ and A_2_” represents the normalized amplitudes of two different populations with different exposure to the solvent. The other parameters “T_1_, T_2_” represents the two bleaching time constants. In the plots, the time “*t*” (the *x* axis) is represented in terms of frame numbers (which also is the step length). A comparison of the bleaching time constants (i.e., step lengths) and amplitudes for all the system is given in Table 1. We note that not all the systems required a second component. We also note that the step-length distribution shows a rising component at initial times (which is especially prominent for the rh-PE data), which has been ignored in these fits. This initial rise is possibly an artifact of unknown origin. Alternatively, this may also indicate a bleaching rate that increases with time. We note that photoexcitation of fluorophores acts as a source of the bleaching agent [^1^O_2_]. This is a diffusion coupled reaction in a combined 3D/2D system, and can be a complicated function of time. However, this possibility has been ignored in the rest of this manuscript, as it is unlikely to affect our inferences.

**TABLE 1:** Fitting parameters of the step length distribution. The normalized amplitudes (A and B) and the corresponding bleaching time constants (in units of frame number, each frame 90 ms) derived from the exponential fit. For each sample type, the first and second row corresponds to the parameters obtained without and with 5 mM tryptophan respectively. Error bars are error of fitting.

Sample	Amplitude 1 (A)	Bleaching time constant 1 (frame number) (a)	Amplitude 2 (B)	Bleaching time constant 2 (frame number) (b)
rh-B PE in PPC bilayer	**0.34 ± 0.08**	**183.0 ± 37.8**	**0.65 ± 0.11**	**359.4 ± 45.0**
	**0.24 ± 0.17**	**635.4 ± 106.8**	**0.75 ± 0.24**	**636.0 ± 90.6**
Lipidated tris rhodamine-b	**1.00 ± 0.00**	**165.9 ± 32.9**		
peptide	**1.00 ± 0.00**	**312.7 ± 48.4**		
hIAPP monomer	**0.43 ± 0.28**	**382.8 ± 71.4**	**0.56 ± 0.14**	**211.8 ± 51.6**
	**0.25 ± 0.16**	**424.2 ± 177.6**	**0.75 ± 0.54**	**263.6 ± 66.21**

**FIGURE 7:**
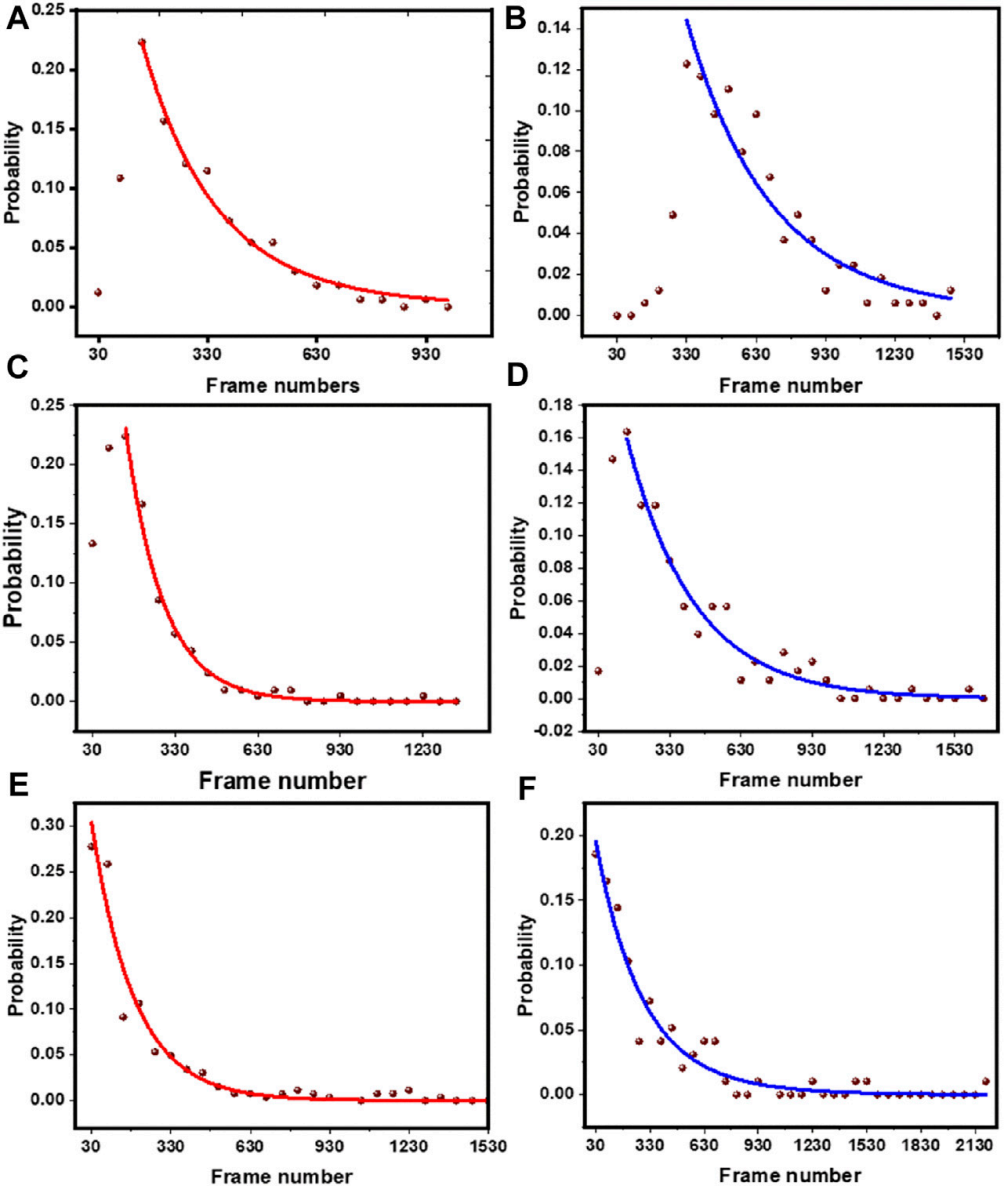
Exponential fits of the step length distribution before and after tryptophan quenching. Rh-PE containing bilayer without tryptophan **(A)** and with 5 mM tryptophan **(B)**, lipid tailed tris rhodamine-B labelled peptide without tryptophan **(C)** and with 5 mM tryptophan **(D),** N-terminal rhodamine-B labelled hIAPP without tryptophan **(E)** and with 5 mM tryptophan **(F)**. Brown dots, data; red (blue) line, distribution before (after) the addition of 5 mM tryptophan.

For a system, if a subpopulation of fluorophore is exposed to the solvent, the bleaching time constant will get shifted to higher values upon tryptophan addition. On the other hand, if the subpopulation is buried, then it will not be affected by tryptophan. In the case of rh-PE in PPC 111 bilayer, the bleaching time constants shift significantly after tryptophan addition (Figures 7A,B). For rh-B PE in PPC bilayer, the fluorophore is in the headgroup which makes it accessible to the tryptophan in solution. This exposure is reflected in the significant change in the bleaching time constant. We also note that the two time constants represent heterogeneity in the population, and is consistent with the steady state photobleaching results which indicate a buried population (Figure 1D). Upon tryptophan addition, the two components essentially became the same. For the lipidated tris-rhodamine-B labelled peptide (calculated for the final step only) (Figures 7C,D), we observed only a single time constant, but it showed a considerable tryptophan induced effect. The hIAPP system showed two time constants and consequently, a heterogeneity in the population. However, the tryptophan-induced shift in the time constants was much lower for hIAPP, indicating a much lower exposure of the fluorophores than the previous two cases (Figures 7E,F). This suggests that the N-terminus of IAPP is the most inaccessible among the all the three molecules studied here, and has at least two different classes of conformations in the membrane. We think that the small changes in the relative fraction of populations reflect the heterogeneity in the tryptophan-rhodamine complexes as described before (Vaiana et al., 2003). We therefore infer that smPB coupled to fluorescence quenching can not only distinguish between molecular assemblies on the basis of their solute exposure, but can also report the heterogeneity in the conformation of a single type of oligomer/molecule.

## 4 Discussion and Conclusion

Single molecule photobleaching (SMPB) can assess the stoichiometry of individual particles based on their bleaching trajectory. We show that combining smPB with fluorescence quenching can increase the step-length of photobleaching, which can in turn measure the relative exposure of different parts of a protein at the level of individual molecules.

We established the basis of our Q-SLIP technique with ensemble measurements, using both solution measurements (Figure 1) and confocal imaging of lipid bilayers (Figure 2). Solution measurements showed that both static and dynamic quenching were in operation, but static quenching had the major effect. If the static quenching originated from stable ground state complexes, then only the dynamically quenched population would be observable by fluorescence, and tryptophan would only cause a minor effect. However, confocal microscopy experiments showed that tryptophan (quencher) can substantially enhance the photostability of rhodamine-B in a lipid bilayer system (Figure 2). This indicates that the static quenching is due to a reversible complex formation, but with a relaxation timescale much longer than the fluorescence lifetime.

The tris-rhodamine labelled KSQKTTKI peptide with a lipid tail served as a model for multimeric protein complexes, where the number of monomers in each particle can be determined by the number of photobleaching steps (Figure 4). The hydrophilic peptide part of the molecule was expected to induce all the three rhodamines to be in similar aqueous environment, while its lipid tail remained anchored to the lipid bilayer. Indeed, our results showed that all the rhodamine molecules in the peptide are stabilized by tryptophan to approximately the same extent (Figure 4C). This experiment verified that fluorophores in similar environments had similar increase in the photobleaching step-length, establishing the robustness of our technique. We also showed, by restricting diffusion in a PVA matrix, that it is indeed the solution-mediated interaction between tryptophan and rhodamine that is responsible for the increase in step length (Figure 4D).

We then extended this technique to the clinically important peptide hIAPP (Figure 5). We found here that the relative increase in step length was much less compared to our standard tris-rhodamine-B lipid-tailed peptides. This implied that a fluorophore at the N-terminus of the membrane-attached IAPP is much less exposed compared to the other two molecules. The power of single molecule approach manifested itself when the underlying step-length distribution was analyzed (Figure 7). We saw that the tris-rhodamine labelled peptide could be fitted to a single component which got shifted to a larger bleaching time constant upon addition of tryptophan (Figures 7C,D). hIAPP contained two populations, with the minor one being more solvent exposed (Figures 7E,F).

**FIGURE 8:**
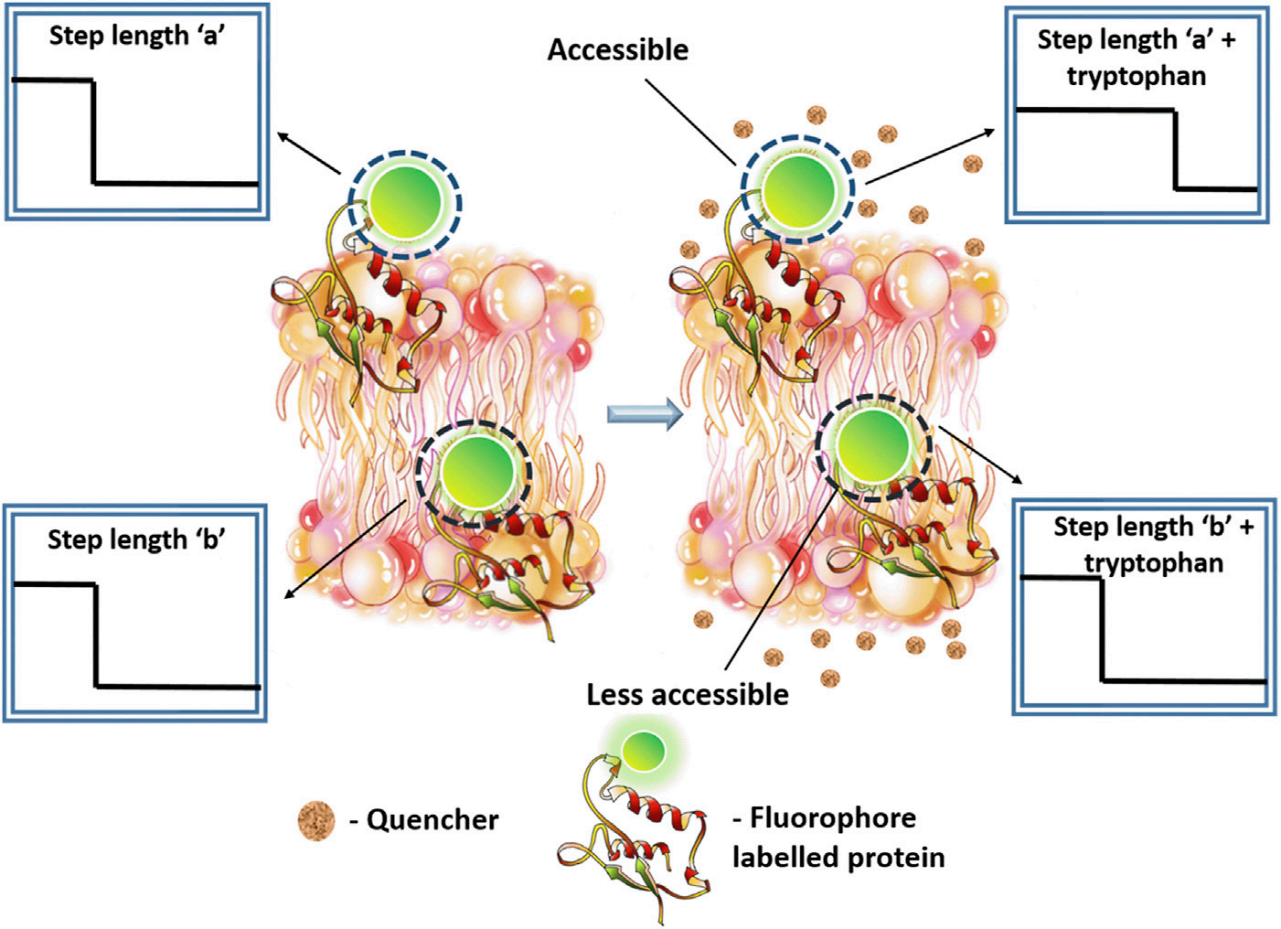
Diagrammatic representation of the final finding from the study. Q-SLIP can probe the relative exposure of any fluorescently labelled part of a membrane protein to an externally added quencher molecule. In the figure, two membrane proteins are shown containing a single label each. Upon interaction with tryptophan, the protein which contains an exposed fluorophore tagged region. **(A)** shows an increase in step length. The other protein. **(B)** which is somewhat less accessible does not show substantial change in bleaching step length.

In summary, we have demonstrated a single molecule technique Q-SLIP that has the power to examine solvent exposure of individual protein molecules. This is especially powerful for examining the different constituents of a multimeric complex, as individual photobleaching steps from different monomeric units can in principle be separately analyzed. The overall finding has been diagrammatically represented in Figure 8. Thus, Q-SLIP can be an effective tool for determining the stoichiometry and the conformation of any monomeric or multimeric membrane protein.

## Data Availability

The raw data supporting the conclusion of this article will be made available by the authors, without undue reservation.
