# Supplementary material for: Single Molecule Measurements of the Accessibility of Molecular Surfaces
Source: Front Mol Biosci. 2021 Dec 1;8:745313. doi: 10.3389/fmolb.2021.745313 (PMC8672140; doi:10.3389/fmolb.2021.745313)
Supplement: Supplementary file 2 [file DataSheet1.docx]

**Supplementary Information**

**SI-1) Mass spectrum of the labelled peptides used for single molecule photobleaching (smPB) experiment**


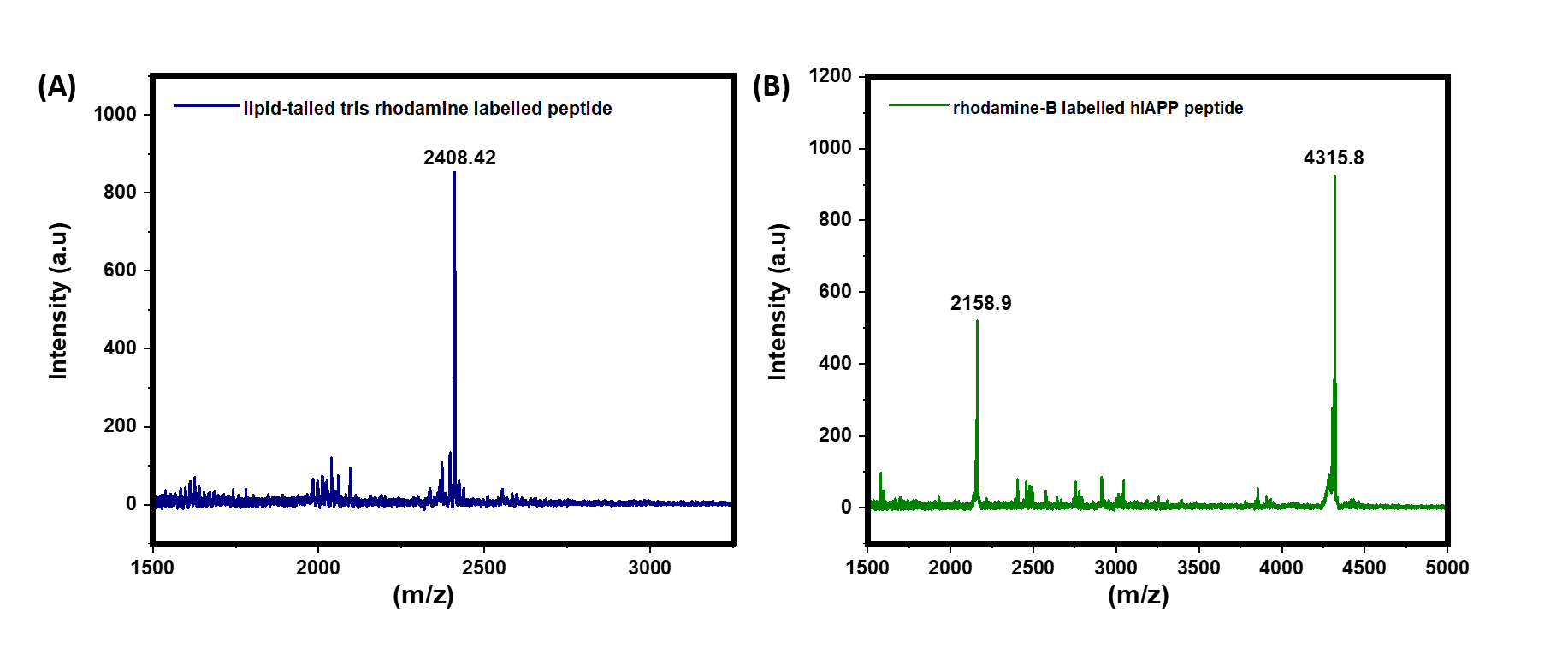


Fig SI-1: MALDI spectra of (A) lipid-tailed tris-rhodamine labelled peptide KSQKTTI, and (B) rhodamine labelled hIAPP peptide.

**SI-2) Tryptophan quenching of 5(6)-carboxyfluorescein**

To check whether tryptophan can be used as a quencher in general for other dyes, we performed steady state and lifetime quenching measurements of 5(6)-carboxyfluorescein with tryptophan. A 200 nM solution of 5-carboxyfluorescein was treated with a range of tryptophan concentrations (up to 50 mM). Corresponding lifetime and intensity were measured and represented as Stern-Volmer plots.

The dynamic quenching data was fitted with the Stern-Volmer equation (Fig-SI-2 (A))

$$\frac{\boldsymbol{\tau}_{\boldsymbol{0}}}{\boldsymbol{\tau}}\boldsymbol{=1+}\boldsymbol{k}_{\boldsymbol{q}}\boldsymbol{\tau}_{\boldsymbol{0}}\left[ \boldsymbol{Q} \right]$$

From the plot, the bimolecular quenching constant was calculated to be 3.17×10^9^ M^-1^s^-1^.


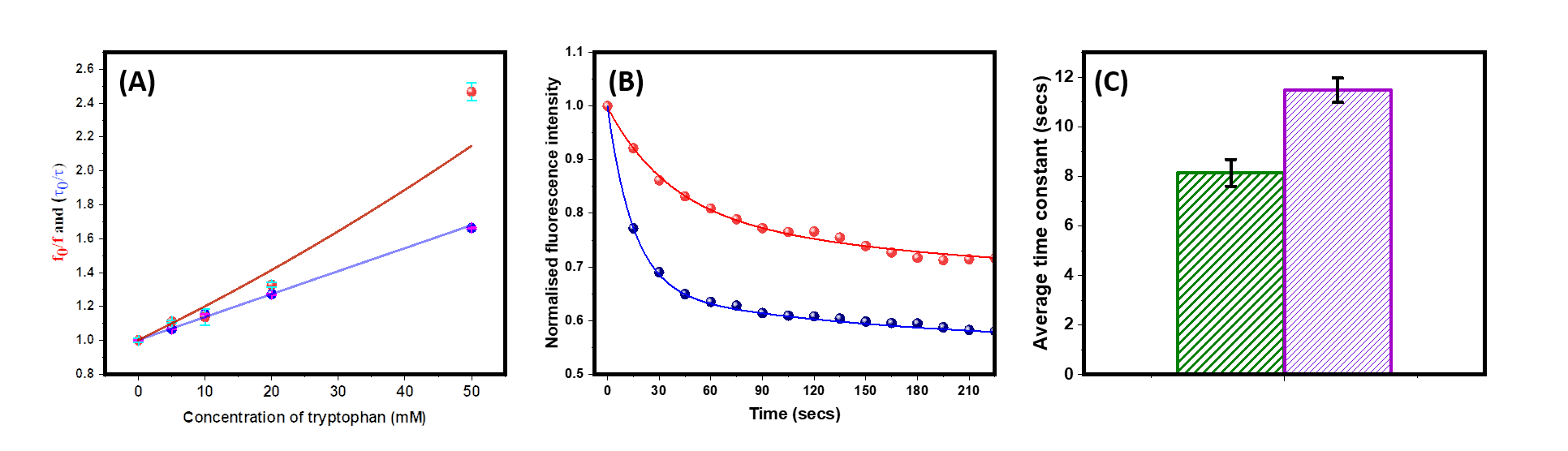


Fig SI-2: Tryptophan induced photo stability in 5(6)-carboxyfluorescein, (A) steady state (red trace) and lifetime quenching measurements (blue trace) of fluorescein with tryptophan, (B) Bi-exponential fit to the confocal bleaching decay of fluorescein in presence (red trace) and in absence (blue trace) of 5mM tryptophan, and (C) The average bleaching time constant obtained from the fits (green – without tryptophan, purple – with tryptophan). All the data points are represented as mean ± SEM**.**

Since the steady-state quenching values deviated significantly from the dynamic quenching data, it was clear that additional quenching process is there. We fitted the steady state quenching data with a modified Stern-Volmer equation

$$\frac{\boldsymbol{F}_{\boldsymbol{0}}}{\boldsymbol{F}}\boldsymbol{=(1+}\boldsymbol{K}_{\boldsymbol{s}}\left[ \boldsymbol{Q} \right]\boldsymbol{)(1+}\boldsymbol{K}_{\boldsymbol{q}}\boldsymbol{\tau}_{\boldsymbol{0}}\left[ \boldsymbol{Q} \right]\boldsymbol{)}$$

Here, K_s_ represents the static quenching constant. From the fit, the value of Ks obtained was 5.6 ± 1.8 M^-1^. This showed that both the static and dynamic quenching are involved in tryptophan induced quenching of 5-carboxyfluorescein.

To check whether tryptophan induced quenching in 5-carboxyfluorescein also induces photo-stabilization, we performed confocal bleaching experiments. A fluorescein labelled lipid, DOPE (1,2-dioleoyl-sn-glycero-3-phosphoethanolamine-N-(carboxyfluorescein ammonium salt) was used as a fluorescent marker for preparing a lipid bilayer of PPC 111 (labelled:unlabelled peptide ratio is 1:60). Fig SI-2 (B) shows the normalized bleaching trajectory with and without 5 mM tryptophan solution (red and blue traces respectively). The Trajectories were fitted for the first 15 frames with a bi-exponential equation,

$$\boldsymbol{y= A}_{\boldsymbol{1}}\boldsymbol{e}^{\left( \frac{\boldsymbol{-t}}{\boldsymbol{T}\boldsymbol{1}} \right)}\boldsymbol{+}\boldsymbol{A}_{\boldsymbol{2}}\boldsymbol{e}^{\left( \frac{\boldsymbol{-t}}{\boldsymbol{T}\boldsymbol{2}} \right)}\boldsymbol{+}\boldsymbol{y}_{\boldsymbol{0}}$$

and the average bleaching time was calculated using the equation,

$$\frac{\left( \boldsymbol{A}_{\boldsymbol{1}}\boldsymbol{T}_{\boldsymbol{1}}^{\boldsymbol{2}}\boldsymbol{+}\boldsymbol{A}_{\boldsymbol{2}}\boldsymbol{T}_{\boldsymbol{2}}^{\boldsymbol{2}} \right)}{\boldsymbol{A}_{\boldsymbol{1}}\boldsymbol{T}_{\boldsymbol{1}}\boldsymbol{+}\boldsymbol{A}_{\boldsymbol{2}}\boldsymbol{T}_{\boldsymbol{2}}}$$

The average bleaching times obtained were 8.14 ± 0.54, and 11.48 ± 0.49 before and after adding 5 mM tryptophan respectively. These are represented in fig SI-2 (C). The green column is before adding tryptophan, and violet column after adding tryptophan. The values are all mean ± SEM. Thus, the percentage stabilization ( ̴41%) is much less than the stabilization for tryptophan. This can be attributed to the small population of labile complex formation in this case.

**SI-3) Brightness analysis and optimization of quencher concentration**

Since Q-SLIP is based on introduction of non-radiative decay processes to an excited state of a fluorophore (so that its triplet state population is reduced), it would have an impact on the average brightness of the fluorophore. Since in case of rhodamine and tryptophan, we have seen that the contributing factor is mainly labile ground state complex formation and dynamic quenching, it implies there should be a reduction in the brightness of the fluorophore. This reduction in brightness should be correlated to the increase in step length for each fluorophores as the non-radiative decay processes form the basis of increase in photostability.

As already mentioned in the manuscript, for the lipid-tailed tris-rhodamine labelled peptide, the step lengths increase by a factor of 2. By measuring the brightness of these three rhodamines (calculated from the background to the maximum intensity for each step), we see each step shows a decrease in brightness by approximately 2 times upon addition of 5 mM tryptophan. As shown in fig SI-3 (A), the reduction in brightness are by factors of 2.13 ± 0.51, 2.21 ± 0.55, and 2.31 ± 0.79 respectively.


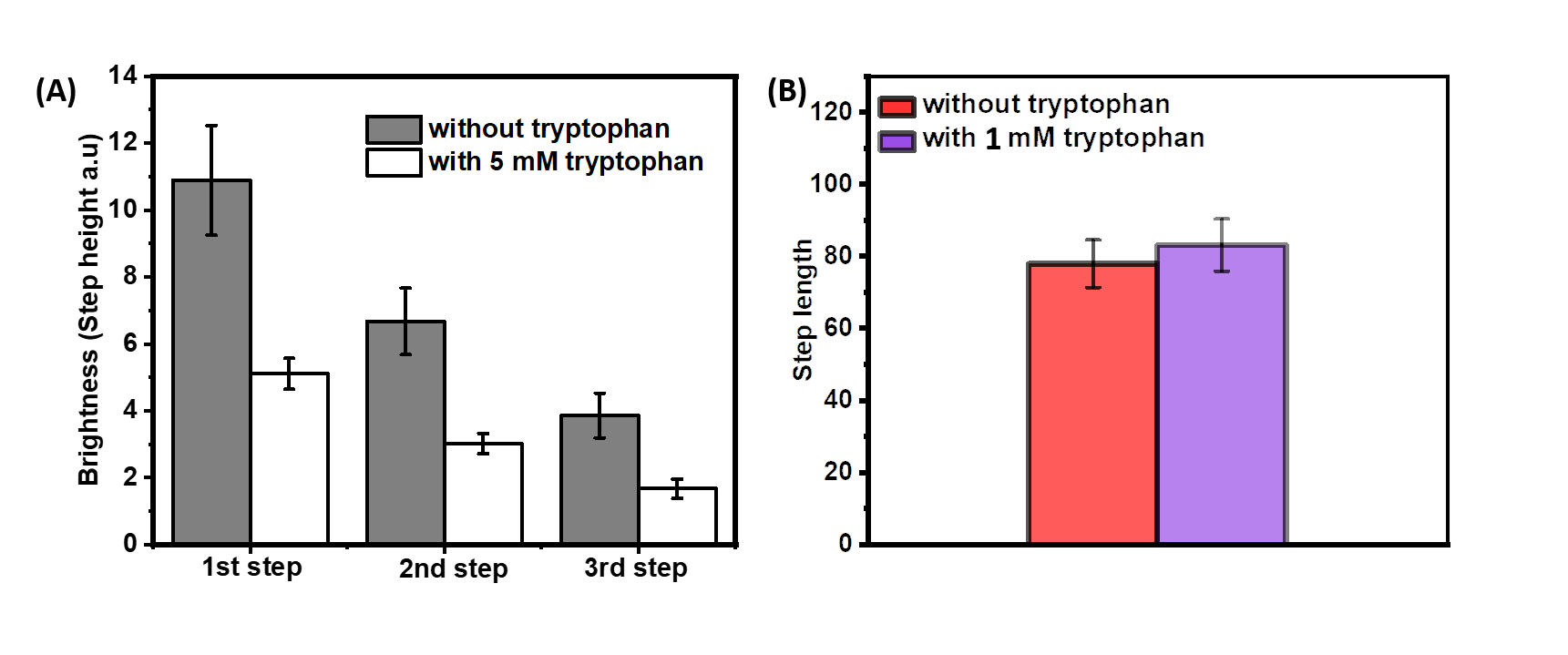


Fig SI-3: (A) Step height (brightness) analysis of the three steps of tris rhodamine labelled lipidated peptide before and after addition of 5 mM of tryptophan, and (B) step length change for N-terminus rhodamine labelled hIAPP with 1 mM tryptophan.

Also, we checked whether the effect of step length increase can concentration dependent. At lower concentration of tryptophan, although the brightness was more, tryptophan induced increase in step length was also reduced. Fig SI-3 (B) shows the no effect of 1mM tryptophan on the step length of hIAPP monomer which otherwise shows a little but significant change with 5 mM tryptophan.
